# The Hidden Role of Community Pharmacy Technicians in Ensuring Patient Safety with the Use of E-Prescribing

**DOI:** 10.3390/pharmacy3040330

**Published:** 2015-11-25

**Authors:** Olufunmilola K. Odukoya, Loren J. Schleiden, Michelle A. Chui

**Affiliations:** 1 Pharmacy and Therapeutics, School of Pharmacy, University of Pittsburgh, 3501 Terrace St, 720 Salk Hall, Pittsburgh, PA 15213, USA; E-Mail: ljs24@pitt.edu; 2 Social & Administrative Sciences, School of Pharmacy, University of Wisconsin-Madison, 777 Highland Avenue, Madison, WI 53705, USA; E-Mail: michelle.chui@wisc.edu

**Keywords:** pharmacy technicians, electronic prescribing, e-prescribing, patient safety, community pharmacies

## Abstract

Objectives: It has been reported that supportive personnel, such as pharmacy technicians, are key participants in the use of health information technology. The purpose of this study was to describe how pharmacy technicians use e-prescribing and to explore the characteristics of technicians that support pharmacists in ensuring patient safety. Methods: This was a qualitative study that used observations, interviews, and focus groups to understand the role of pharmacy technicians in e-prescribing. Fourteen pharmacy technicians and 13 pharmacists from five community pharmacies participated. Observations lasted about nine hours in each pharmacy. Follow-up interviews and two separate focus groups were later conducted. Observation field notes and audio recordings were transcribed and thematically analyzed. Results: Pharmacy technicians were primarily responsible for all steps leading up to pharmacist review of the e-prescription and dispensing of medications to the patient. Technician characteristics, including experience, certification status, and knowledge of appropriate medication use, were reported as important factors in supporting a pharmacist’s role in ensuring patient safety with the use of e-prescribing. Conclusion: Study findings indicate that pharmacy technicians have an important role in supporting pharmacists to prevent medication errors. Certain characteristics of pharmacy technicians were identified with the potential to improve the e-prescription medication dispensing process and decrease patient harm through the identification and resolution of errors.

## 1. Introduction

Medication errors are a significant problem in healthcare settings [1,2,3]. Medication errors can lead to suboptimal outcomes in patients, including increased cost [3], longer hospitalization [4], injury [5] and death [6]. One form of health information technology (health IT) that has been promoted to reduce medication errors in various healthcare settings is electronic prescribing (e-prescribing) [7,8,9]. E-prescribing systems allow physicians to electronically generate and transmit prescriptions to community pharmacies. In the United States, e-prescriptions are entered by the prescriber and transmitted to a transaction hub, which links the prescriber, preferred pharmacy system, and pharmacy benefits manager. E-prescribing presents an alternative to more traditional methods of transmitting prescriptions via phone, fax, or paper. E-prescribing system use has seen tremendous growth over the past decade, from 29 million e-prescriptions generated in 2007 to 1.20 billion in 2014 [10]. In community pharmacies that are primarily responsible for processing prescriptions, it is estimated that prescription error rates are as low as 0.23% and as high as 11% [11,12,13,14,15,16,17,18]. Despite the emergence of e-prescribing as a major method of sending prescriptions from the prescriber offices directly to community pharmacies, there is evidence that e-prescribing does not reduce prescription error rates [19].

E-prescriptions sent to community pharmacies are often first received and processed by pharmacy technicians [20]. However, little is known about the role of pharmacy technicians in using e-prescribing and their contributions to preventing medication errors in community pharmacies. Pharmacy technicians are supportive staff who can work in a variety of pharmacy settings, such as community, hospital, and assisted living facility pharmacies. Pharmacy technicians at community pharmacies are typically responsible for all of the steps in preparing a medication to be reviewed by a pharmacist and subsequent dispensation to the patient [21].

There is compelling evidence of the impact of similar types of medical supportive staff, such as nurses, physician assistants, and medical assistants, in making important contributions to improving patient safety in healthcare settings [22,23,24,25,26,27,28,29,30]. Studies of the characteristics of supportive staff have given insight into their significance in improving patient safety, including reduction of healthcare-associated infections through implementation of a unit-based quality nurse [30], association of reduced failure to rescue with an increased nurse to patient ratio [22], association of hospital nurse educational level and surgical patient mortality [23] and the decrease of the length of stay and patients who leave without being seen at emergency departments through the addition of a physician assistant as a triage liaison provider [24].

While there is convincing evidence of the significance of other types of medical supportive staff in improving patient safety [25,26,27,28,29], there has been little attention given to the role of pharmacy technicians in using health IT, such as e-prescribing to improve medication safety. It has been reported that about 11% of e-prescriptions sent to community pharmacies from prescriber offices have medication errors [19]. Since pharmacy technicians are the first personnel in community pharmacies to handle processing of prescriptions and the high rate of e-prescription errors encountered, it is important to explore how the characteristics of pharmacy technicians influence patient safety. Previous research on e-prescriptions in England has investigated types of errors encountered in community pharmacies along with implications for increasing efficiency in the process, such as keeping databases used by dispensing systems updated [31,32]. Examining the processes involved in preventing, identifying and resolving e-prescription errors in the United States could inform efforts at community pharmacies to prevent e-prescription errors from happening, identify e-prescription errors when they do happen, and fix e-prescription errors in an efficient and effective manner.

## 2. Objective

The purpose of this study was to describe how supportive personnel in community pharmacies, specifically pharmacy technicians, use e-prescribing and the characteristics of technicians that ensure medication safety.

## 3. Methods

Pharmacist technicians and pharmacists at five Southwest Wisconsin community pharmacy sites (two chain pharmacies, three independent pharmacies) with previous collaborative relationships were invited to participate in this study from October 2012 to April 2013. Participants gave informed consent and were remunerated $50 each for their participation. Approval for this research was granted by the University of Wisconsin-Madison Institutional Review Board. This research was part of a larger study that explored e-prescription error recovery in community pharmacies, and additional details of the methods can be found in previous publications [20,33]. Data were collected using three different methods: observation, interviews and focus groups. These methods were used to understand how technicians handled e-prescriptions and prevented associated medication errors.

## 4. Direct Observations

Direct observations were used to understand how pharmacy technicians processed e-prescriptions and dealt with any medication errors as they were encountered. Pharmacy technicians were observed in four-hour intervals on two separate days, and these intervals typically took place on weekdays during the hours of 8 a.m. and 8 p.m. Observations on the first day took place during the early hours of operation, and observation on the second day took place during the later hours of operation. Two researchers with prior patient safety research experience (one pharmacist and one human factors engineer) observed participants as they processed e-prescriptions. Participants were instructed to perform their regular duties as usual and to notify the observing researcher when they encountered a medication error. Researchers took extensive field notes on how e-prescriptions were processed by pharmacy staff, which were transcribed within 24 hours of the observation period. A total of 26 pharmacy personnel participated in direct observations, including 15 technicians and 11 pharmacists. All direct observations were conducted before any participants were interviewed. Data gathered from direct observations were used to inform interviews with participants.

## 5. Interviews

Semi-structured interviews were administered in order to collect additional information on how e-prescriptions were processed by pharmacy technicians, including the process of dealing with medication errors when they were encountered. An interview guide was developed to provide predetermined questions, prompts, and probes to help elicit additional information about how e-prescriptions were processed and how medication errors were dealt with. Questions in the interview guide were designed to be neutral and open in order to keep from educing socially desirable responses [34]. Questions were also examined for content validity by five experts, including four pharmacists and one human factors engineer.

Two researchers conducted the interviews, which lasted approximately one hour each, with one researcher facilitating the interview while the second researcher took notes and provided support in asking questions and prompting for more information from participants. Nine pharmacy technicians and eleven pharmacists were interviewed. Of the 20 interview participants, 17 had also participated in the direct observations. Each interview was audio-taped, and all audio recordings were professionally transcribed. Interview transcripts were verified for accuracy by listening to eight audio-recordings of interviews (40%). The interview transcripts and data analysis plan were discussed by three researchers. Data obtained from direct observations and interviews were used to inform the focus groups with participants.

## 6. Focus Groups

Two separate focus groups were conducted: a technician focus group and a pharmacist focus group. Both focus groups took place after all interviews were completed, and both were facilitated by the first author. A focus group guide was created by triangulating and summarizing direct observation and interview data from all pharmacies and was used to help direct focus group discussions. This guide gave participants the chance to: (1) provide additional information not captured during observations and interviews; (2) learn about data gathered from other pharmacies; and (3) verify the interpretations of and to give the data collected. The guide also sought to examine in more depth the role of technicians in processing e-prescriptions and handling medication errors. Questions and summary data documents were reviewed by three researchers and presented to participants in the form of summaries of strategies used to process e-prescriptions and handle medication errors from all pharmacies. Focus group participants were emailed the focus group questions and documents one week prior to the focus group session.

Four technicians participated in the technician focus group held eight weeks after the last interview (March 2013). Eight pharmacists participated in the pharmacist focus group held in early April 2013, with one pharmacist joining the focus group via telephone. All focus group participants had previously participated in the individual interviews. Each focus group lasted approximately 90 minutes. Researchers held a debriefing session immediately after completion of the first focus group (technician focus group) to review the focus group questions and discuss changes needed for participants in the second focus group (pharmacist focus group). Both focus groups were tape-recorded and transcribed by a professional transcriptionist. The first author verified the accuracy of the transcription process by listening to both focus groups. A comprehensive summary, including transcripts of audiotapes, anecdotal notes and field notes, was generated following the completion of both focus groups.

## 7. Data Analysis

Observation field notes, interview transcripts and focus group questions were imported into the qualitative analysis software NVivo 10 and subjected to content analysis [35]. Two researchers reviewed field notes and transcripts line by line to identify sections of the text that were assigned codes. Codes were further reviewed to identify patterns and themes that were summarized into higher-level themes. The third author reviewed these themes, and the research team met to address disparities and inconsistencies within the data and to create final descriptions of the study findings.

## 8. Results

### 8.1. Characteristics of Technicians and Pharmacies

On average, participating pharmacies processed about 120 to 400 e-prescriptions daily. Each pharmacy generally had about two to five technicians on staff daily. The age range of technicians was 22 to 59 years old, and their experience in pharmacy practice ranged from 3 to 30 years. A few of the technicians had received formal technician training and were certified technicians.

### 8.2. E-Prescription Processing: Pharmacy Technician’s Role in Ensuring Safety

**Figure 1:**
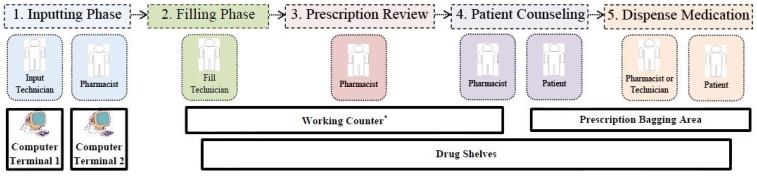
E-prescription processing and layout of community pharmacies. Notes: * Working Counter is used for inputting, filing, checking, and dispensing medications. Holds tools used for prescription processing, such as prescription dispensing bins, calculators, labels and computers.

Technicians were primarily responsible for processing e-prescriptions in community pharmacies. The general process for processing e-prescriptions in community pharmacies is shown in Figure 1. In the initial step of e-prescriptions processing, a notification was received that an e-prescription had been sent from the prescriber’s office directly into the e-prescribing queue in the pharmacy’s computer system. In some of the study pharmacies, the e-prescription was then printed by the technician and the information either manually re-inputted or electronically auto-populated into the patient’s profile in the pharmacy system (the inputting phase). For pharmacies in which e-prescriptions were first printed, technicians gave three main reasons for printing e-prescriptions: (1) to prevent errors during inputting of information; (2) for easy verification of information; and (3) for record keeping purposes. The e-prescription information, such as a patient’s or physician’s information (name, date of birth, address, telephone number) and drug information (name, strength, dosing directions, quantity), did not always enter the pharmacy system directly. Such information was sometimes manually typed into the patient’s prescription profile in the pharmacy system.

Pharmacist: “The primary responsibility for processing prescription is the technicians. So generally they will receive the notification that there is an e-prescription in the queue, and then they will process it in the computer. And after they processed it, a paper version of the e-prescription will print out to be used as a check during the checking process and filling process of the prescription. And then as the pharmacist, we’ll receive the paper version that’s been printed off and use that to check the accuracy of the filled prescription.”

Technician: “We just don’t fill them from the e-prescription…The directions, if you do fill it off of [the computer], without printing it out, all those directions will be on there. So if you, let’s say, don’t catch the two different directions on there, both directions are going to be on that prescription for that patient unless you would delete it or change it. So nothing is, you know, there isn’t one thing that would probably ever be right if you were to fill it off of an e-prescription.”

Technician: “And so I print out the hard copy, and then I go through and, I’ll actually use the computer, I’ll take the hard copy and then just use that to compare against what comes up on the screen for the patient and everything… Then you look at the physical prescription there. And then you look down at the doctor’s name, make sure that that matches up. And then I usually give the prescription one more look-over. I’ll circle the date on the front of the prescription.”

Technicians also played a significant role in detecting e-prescription errors during inputting and filling through verification of prescription information. The input technician typically initiated the inputting of the e-prescription into the pharmacy system; however, in one pharmacy, the pharmacist was responsible for inputting e-prescriptions into the pharmacy system. One participant reported that input of an e-prescription was done very fast and took about 30 seconds, and this was also witnessed by researchers during the observation periods.

Technician: “Most of the time, they enter so fast, which is a nice thing. I mean, it takes you like 30 seconds to enter an e-prescription. So you can do them fast.”

During the inputting phase, the input technician or pharmacist verified the e-prescription information as it was being entered into the patient’s profile and then printed the appropriate labels. During inputting, the patient’s medication profile was sometimes reviewed to determine if the prescription was appropriate for the patient. For example, the inputting technician verified patient allergies, how often the patient was receiving the prescriptions and compared past dosing regimen with the e-prescription being inputted.

Technicians: “The e-prescriptions are seen by the technician first.”

The filling phase (second check) was the next step; this was done mainly by the designated filling technicians. The input technician placed the printed e-prescription and medication label in a dispensing bin that was then passed down to the fill technician. The fill technician was responsible for picking medications from the shelves or dispensing robot, counting medications, and placing them in dispensing bottles, attaching appropriate labels, and ensuring that the number of counted pills filled matched the printed e-prescription label. Once the prescriptions were filled and labeled, they were put into specific colored dispensing bins and passed down the workflow for final check and review by the pharmacist. Different colors of dispensing bins were used to notify the pharmacist of the type of prescription or if the patient was waiting to pick up their medications. However, all five pharmacies used different colors to designate prescription information. For example one pharmacy used the pink dispensing bins to indicate that patients were waiting in the pharmacy to pick up the prescriptions and used green colored dispensing bins to indicate that an e-prescription had an error or an unresolved problem. Another pharmacy used white bins to indicate that the e-prescription was a refill medication, and a red bin meant the patient was waiting to pick up the medication.

Technician: “What we do also is when we fill the prescription, then when we go, well, we fill it in the computer, and then we actually count out the pills ourselves and then pass it down to the pharmacist. But what we do when we’re filling too, after we’re done, we’ll take the bottle, look at the NDC underline the NDC number, make sure we got the right bottle off the shelf to match your label, and then we circle the quantity and make sure we had counted out the right quantity, so it eliminates the error there.”

(NDC: National Drug Code, a unique number used to identify medications in the United States)

The next phase was the pharmacist overall review of the e-prescription and filled medication (third and final check). Pharmacists were primarily responsible for checking the accuracy of the e-prescription filled and inputted by the technicians; this was commonly described by participants as performing “DUR” (drug utilization review). The pharmacist’s review of the e-prescription consisted of verification of the following: (1) information entered by the input technician; (2) the corresponding medication filled by the fill technician; (3) drug-drug interactions; (4) dosage changes; and (5) overall medication profile history of the patient. Finally, pharmacists bagged the prescription and proceeded to counsel the patient before dispensing the medication to the patient (patient counseling and dispensing of medication).

### 8.3. What Pharmacy Technicians Do to Support Pharmacists’ Role in E-Prescribing

Technicians performed a variety of tasks when processing e-prescriptions to enable the pharmacist to be more efficient in making sure medications were accurately dispensed. For instance, technicians memorized pharmacist preferences, assisted the pharmacist to look up drug information using online tools, and performed a second check to drug or patient information inputted by the pharmacist into the pharmacy’s e-prescribing system.

Of particular importance for technicians was having a general awareness of what was going in the pharmacy. For instance, technicians were usually the first set of eyes to review e-prescriptions and to detect errors. A primary role of technicians in e-prescription processing was ensuring patient safety by detecting medication errors, because most were caught during the inputting of the e-prescription information. One technician expressed that technicians were the first individuals in the pharmacy to review e-prescription for errors, and it was their job to detect errors.

Technician: “Well, a lot of the times, you’re like the first, well, at least I’m the first person to see it if I’m sitting there and entering whatever. So I think it’s just my job if I’m there like first, or I’m late instead of passing it on and making the second technician and finally the pharmacist finds it, and then the patient is there already. So I think like since I’m the first set of eyes, it’s my job to find errors first and to be looking for them.”

Technicians made sure to communicate with the pharmacist on shift about e-prescription errors that were detected. Technicians were also proactive in using a variety of strategies, such as colored bins or flags to distinguish that an error was suspected, to ensure the pharmacist performed a double check on an e-prescription that they suspected had an error. Pharmacy technicians also examined the patient’s medication profile prior to the pharmacist’s review of the e-prescription so as to be proactive in alerting the pharmacist of a medication error. Technicians also served as second check or a “second set of eyes” to look over e-prescriptions that had been previously reviewed by a pharmacist for things like inputting or spelling errors.

Technician: “I do three checks along the way. Like I check the NDC when I’m grabbing it off the shelf. I check the NDC when I’m pouring the excess back into the bottle. And when I set it down, I check NDC and quantity that I’ve just counted, and I figure I’m doing it three times along the way. Even If I miss one of those, I’m still doing it twice, so just, you know, having a very good system and like trying to stick to it is really good for reducing errors.”

Technician: “I mean, and that may just be an entering error rather than a prescription error, but I’m the spell check for the pharmacy too, so I just make sure that there’s nothing weird in there. And, I mean, we have short codes for entering stuff in, because the way our software works, you only get so many characters. Like the characters that can be on the label are greater. The characters will let you type in the field, so they have short codes that are separated by semicolons. If they like don’t hit the semicolon or something, then you just end up with the short code in it rather than the actual words. So I keep an eye out for that and make sure that everything is actually, you know, real words and not abbreviations.”

The goal of reviewing prescription was to make sure the medication order was accurately inputted into the pharmacy system and that patient or drug information matched up correctly with the intention of the prescriber. Technicians’ knowledge of appropriate medication use was useful to the pharmacist because the technician could be proactive about calling the prescriber to address errors on the e-prescriptions, especially when the pharmacist was busy. Some pharmacists gave technicians autonomy to contact prescribers directly without asking the pharmacists, particularly when they perceived that the e-prescription error was minor and would not be complicated to resolve. In these cases, the technician was able to reduce the pharmacist’s workload and prevent the task from interrupting the pharmacist.

Technicians also tried to keep abreast of patient information, such as drug allergies, to easily detect errors. Overall, a high attention to detail was important for technicians to contribute to enhancing patient safety.

Technician: “We have one patient that has a yellow dye allergy, so whenever they get a new medication, I, like just on my own, like I just go grab the thing, grab the insert, take a look at all the ingredients and make sure there’s not yellow dye in that.”

During the focus groups, technicians stated that it was helpful to learn about strategies used by technicians in other pharmacies to ensure e-prescribing safety. The focus group served as a learning session to compare the role of technicians in other pharmacies and how useful strategies could be incorporated into their respective pharmacy settings.

Technician: “...if they have other strategies for what they do, it might work for us, just as our strategies might help them.”

Technician: “...it was interesting to learn that the same problems that we deal with…, everyone else, you guys deal with.”

### 8.4. How Do the Characteristics of Pharmacy Technicians Impact Medication Safety with E-Prescribing?

During the pharmacist focus group, pharmacists discussed various characteristics of pharmacy technicians that would have an impact on their ability to detect errors. These included having more pharmacy practice experience, certification as a pharmacy technician, a high level of commitment, critical thinking skills, close attention to detail, and a proactive personality.

Pharmacist: “It’s level of experience, for one. The longer they’ve been in this field or in its employ, that is tremendous. Those who are, those who have been, are certified technicians, have also indicated, you know, a higher level of commitment. I look at that as someone has a higher level of commitment, you know, in wanting to make this and everything more of a career for them. So I think that helps tremendously.”

Pharmacists indicated that having a full-time pharmacy technician on their shift makes their jobs easier, as full-time technicians who deal with working in a pharmacy on a daily basis may have more familiarity with common e-prescribing issues.

Pharmacist: “One thing that makes it a little bit easier is that we typically have a full-time technician on every shift. So there’s somebody that has a lot of experience dealing with e-prescriptions, so they’re familiar with a lot of the common issues. And they’re, they tend to be more experienced technicians as well, so they, they’re helpful at identifying issues or supporting me when I need to address an issue and contact a physician.”

Pharmacists reported that a pharmacy technician being overconfident, rushing or not being well trained could lead to medication errors getting through to the pharmacist or patient.

Pharmacist: ”...while our technicians help us, they can also make a barrier, whether they’re not as well trained, or they are overconfident, or they go too fast, or whatever it is, where they’re not checking their work, and that could be a barrier.”

Pharmacist: “I think one thing that hurts is that you don’t really get a whole lot of training on e-prescriptions. It’s just sort of a, you get used to doing them over time, just practice. So I don’t know that, I don’t know that it’s a whole lot different from other prescription filling that we do though, so. It could just make it a little bit more confusing when you’re working with a newer technician, because they may not be as familiar with the ins and outs of the e-prescribing processing.”

### 8.5. What Can Pharmacists Do to Encourage Valuable Contribution From Pharmacy Technicians?

The quality of interaction between pharmacists and pharmacy technicians was reported as a factor that could enhance pharmacy technicians’ ability to properly handle medication errors. Pharmacists in the focus group reported that trusting technicians to process e-prescriptions and handle any medication errors that might be encountered, empowering technicians to call physician offices to fix medication errors, and reassuring and providing positive reinforcement to technicians could result in further valuable contributions from technicians in processing e-prescriptions. For technicians with different experiences and employment histories, not all technicians will have the same concept of what they can and cannot do within their roles. By providing consistent trust, encouragement, and positive reinforcement, pharmacists stated that technicians may be more confident and knowledgeable in their roles and, thus, more likely to contribute in appropriate situations.

Pharmacist: “...empower the technician to go ahead and do so. In other words, if you let them know that you have the trust, okay, because, you know what, because they earned it, I think that really kind of goes a long way too.”

Pharmacist: “Or maybe it takes a couple times of you giving them the reassurance. Like it’s fine if you want to just go ahead and call on that, or you, yeah, that’s good, that’s a good catch. You know, why don’t you check on it. And then after a few times, then they realize...”

## 9. Discussion

Community pharmacies are healthcare settings where prescribed medications are frequently dispensed to patients. Pharmacists practicing in these settings act as middle men between prescribers and patients to ensure that patients receive the right medications, thus playing a key role in intercepting prescribing errors and ensuring medication safety. Due to increasing workload and prescriptions volume (an estimated 3.3 billion annually) in community pharmacies [36,37,38,39], pharmacy technicians are increasingly playing important roles in the dispensing of medications. Within these settings, pharmacy technicians perform a variety of tasks related to the processing of these prescriptions under the guidance of a community pharmacist [40]. E-prescribing is now the primary way community pharmacies receive prescriptions from physician offices [10]. As e-prescribing becomes more widely used, it is important to examine the role of technicians in ensuring medication safety with this health information technology. Ensuring the safety of e-prescriptions has become paramount, because 11% of e-prescriptions received in community pharmacies have medication errors [19]. Findings from this study highlight the role of pharmacy technicians in processing e-prescriptions in community pharmacies and efforts they make to prevent medication errors from reaching patients.

The findings from this study indicate that all of the steps in the inputting and filling phases of processing an e-prescription were typically conducted by pharmacy technicians prior to verification by a pharmacist. Pharmacy technicians can receive traditional and electronic prescriptions, review for accuracy, prepare orders, package and label medicine, assist patients and maintain patient records. Pharmacy technicians in this study reported that they used a variety of strategies to review prescriptions and to ensure that any medication errors were identified and either: (1) resolved if the error was not complicated; or (2) communicated with the pharmacist if the error was beyond the expertise of the pharmacy technician.

Despite their integral role in processing a prescription, there is still no uniform national requirement for pharmacy technician training. While some pharmacy technicians have completed an educational program to achieve an associate’s degree, diploma, certificate or license, others have been trained on the job [41]. Consistent training of pharmacy technicians through continuing education or taking advantage of on the job learning opportunities could improve their job performance. However, continuing education is not required among all pharmacy technicians, and on the job training may be inconsistent and depend on the availability and ambition of the pharmacy technician. In a recent study, only 16.8% of pharmacy technician respondents reported that, in their most recent medication preparation error, pharmacists provided instruction on how to reduce the chance of a similar error in the future [42]. Findings from our study indicate that pharmacist-technician working relationships can be improved by pharmacists trusting, empowering, reassuring and providing positive reinforcement to help a pharmacy technician to gain confidence and knowledge in his or her roles. Targeting pertinent continuing education topics, taking advantage of possible on the job learning opportunities and promoting confidence and knowledge in their roles could lead to enhanced effectiveness in pharmacy technicians being able to identify and correct medication errors before they reach the pharmacist or patient.

Previous studies have reported that education level, continuing education, training, and related characteristics of other similar types of supportive personnel have been associated with impacting patient safety outcomes [22,23,24,25,26,27,28,29,30]. Considering the important role pharmacy technicians have in processing e-prescriptions, it is reasonable to surmise similar impacts of the characteristics of pharmacy technicians on patient safety outcomes related to medication errors. Pharmacists in this study reported that person-specific characteristics, such as experience, certification, commitment, critical thinking skills, close attention to detail, a proactive personality, and full- *versus* part-time status, impacted how well a technician could perform his or her patient care responsibilities. These associations could have implications on clinical outcomes, such as performance or accuracy in processing e-prescriptions, identification of medication errors, and efficiency in resolving errors when they do occur. The positive implications of optimizing these outcomes include less prescriber, pharmacist and technician time and resources taken to resolve medication errors and fewer medication errors possibly reaching patients and causing harm.

## 10. Study Limitations

The nature of this study was exploratory and only involved five community pharmacy sites in Southwest Wisconsin, the United States. As such, these results may not be generalizable to all community pharmacy sites across the nation and other types of pharmacy settings.

## 11. Conclusions

Pharmacy technicians have been shown to play an important role in the e-prescription process and medication dispensing community pharmacies. Many specific characteristics of pharmacy technicians were reported to be associated with better support of pharmacists in fulfilling their patient care responsibilities and more efficient identification and resolution of medication errors. The true magnitude of these associations with key outcomes, such as productivity and patient safety, are currently unknown. Future research could be done to determine these associations; interventions could be better informed to target potential areas of improvement in an effort to optimize the e-prescription process and robustness of pharmacy technician training to improve medication safety.
